# A numeric-based machine learning design for detecting organized retail fraud in digital marketplaces

**DOI:** 10.1038/s41598-023-38304-5

**Published:** 2023-08-02

**Authors:** Abed Mutemi, Fernando Bacao

**Affiliations:** NOVA Information Management School (NOVA IMS), Universidade Nova de Lisboa, Campus de Campolide, 1070-312 Lisboa, Portugal

**Keywords:** Engineering, Mathematics and computing

## Abstract

Organized retail crime (ORC) is a significant issue for retailers, marketplace platforms, and consumers. Its prevalence and influence have increased fast in lockstep with the expansion of online commerce, digital devices, and communication platforms. Today, it is a costly affair, wreaking havoc on enterprises’ overall revenues and continually jeopardizing community security. These negative consequences are set to rocket to unprecedented heights as more people and devices connect to the Internet. Detecting and responding to these terrible acts as early as possible is critical for protecting consumers and businesses while also keeping an eye on rising patterns and fraud. The issue of detecting fraud in general has been studied widely, especially in financial services, but studies focusing on organized retail crimes are extremely rare in literature. To contribute to the knowledge base in this area, we present a scalable machine learning strategy for detecting and isolating ORC listings on a prominent marketplace platform by merchants committing organized retail crimes or fraud. We employ a supervised learning approach to classify postings as fraudulent or real based on past data from buyer and seller behaviors and transactions on the platform. The proposed framework combines bespoke data preprocessing procedures, feature selection methods, and state-of-the-art class asymmetry resolution techniques to search for aligned classification algorithms capable of discriminating between fraudulent and legitimate listings in this context. Our best detection model obtains a recall score of 0.97 on the holdout set and 0.94 on the out-of-sample testing data set. We achieve these results based on a select set of 45 features out of 58.

## Introduction

Recently, there has been a growth in the use of internet commerce and communication platforms, heightened even more by the COVID-19 pandemic. More than ever before, a sizable portion of the population conducts normal activities online and at home, including work, school, shopping, doctor appointments, and entertainment^1^. Cybercrime and fraud have expanded substantially in line with the widespread use of digital devices and platforms^2^ continuing the pattern of losing the global economy billions of dollars^3^ and jeopardizing community security^4^.

Cybercrime and fraud encompass a diverse range of heinous actions, including phishing, malware, fraudulent e-commerce, romance scams, tech support scams, extortion or blackmail, and denial of service^1^. Additionally, there are instances of credit card theft, money laundering, and plagiarism. Both practices have a detrimental effect on both enterprises and customers, posing significant economic, reputational, and psychological dangers to these entities.

Combating cybercrime and fraud is a time-consuming and costly task since bad actors are always evolving and capitalizing on new chances to exploit the vulnerabilities of existing fraud protection and detection systems. Low development efforts exacerbate the problem further by limiting the sharing of ideas in fraud research. For instance, it makes no sense to explain fraud detection or prevention techniques in the public domain, as this could provide fraudsters with the information necessary to elude detection.

When it comes to addressing cybercrime and fraud, whether through prevention or detection, there are two primary methodologies documented in the literature. Prevention refers to steps taken to avert the occurrence of the acts in the first place. These include intricate designs, personal identity numbers, internet security for online interactions with digital platforms, and passwords and authentication mechanisms for computers and mobile devices^5^. None of these solutions is perfect; frequently, a trade-off between cost (for the business) and discomfort (for the customer) must be made. On the other hand, detection entails recognizing fraudulent acts as soon as they occur^5^. When prevention fails, it becomes material. For example, we can prevent credit card fraud by protecting our cards insidiously, but if the card information is stolen, we must notice the fraud as soon as possible^5^.

There are two conflicting schools of thought when it comes to developing fraud detection and prevention systems. The first is pro-statistical and computational methods, with researchers such as^5–7^ publishing extensively in this area. This school of thought applies statistical tools, including machine learning algorithms, to detect fraud. Classifiers can be trained to discern between the two classes using labeled data (fraudulent and non-fraudulent). In these circumstances, classifiers are fed data from user profiles such as transaction amount, day of week, item category, age, gender, and geography. Those who argue against statistical and computational methods contend that these characteristics are easily fabricated by sophisticated fraudsters^8^. Irani, Pu, and Webb^9,10^ believe that once fraudsters discover that authorities have picked up on their jargon, they can avoid keyword traps by switching to new phrases. The latter school of thought proposes network analysis as an alternative method for developing fraud detection features^8,11^. The concept takes advantage of the connectedness between nodes, often users or items, in a dataset to derive graph-theoretic variables or scores that uniquely characterize nodes. The strategies are predicated on the premise that abnormal users exhibit connection patterns that differ from those of regular users^8^.

We do not officially subscribe to any of these schools of thought in our situation. Rather than that, we argue that the approach to fraud detection should be governed by the problem’s context and influenced further by many stakeholders invested in the goal of reducing fraud instances. As a result, it is vital to build systems that are constantly learning and adapting in order to keep bad actors at bay. Additionally, while we accept that human behavior, social and cultural aspects are key considerations when designing detection and prevention systems^1^, we argue that they must operate in concert with automated processes to rein in the rising trend in fraud cases.

Automation of fraud detection by data mining and machine learning approaches represents a once-in-a-generation chance to significantly lessen the burden on humans while still adjusting to a dynamic fraud environment. In this paper, we emphasize the importance of automation in fraud detection with a machine learning approach to introduce efficient and scalable fraud detection in a domain that is rife with manual processes and inefficient methods such as heuristics and rule-based approaches. We present a framework for machine learning based on an experimental design setting in which we search for the optimal learning algorithm for discriminating between fraudulent and non-fraudulent events.

Our issue is contextualized within the backdrop of organized retail crime (ORC). ORC is defined as the widespread theft of everyday consumer goods from brick-and-mortar establishments, with the stolen goods then resold or fenced to other retailers or individuals via a variety of channels. In the digital age, organized retail thieves have become increasingly savvy, fencing their stolen products using online digital marketplaces. They intend to receive the same benefits as legal vendors from digital platforms, such as increased productivity or efficiency in trading^7^ their stolen products.

Along with other forms of fraud, the economic costs of organized retail crime are substantial and have been increasing at an alarming rate year after year. Retailers lose an average of $719,548 per $1 billion in sales, according to the 2020 National Retail Foundation Organized Crime Survey. These losses are much higher than the $703,320 in 2019 and the $453,940 in 2015. Three in four ORC victims report an increase in ORC in 2020^12^. Retailers believe the increase in ORC-related events is a result of altered shoplifting laws and punishments. ORC has a significant impact on crime statistics and revenue loss; it erodes the viability of retail firms; and it is commonly used to finance other illegal operations^13^. The literature on ORC is sparse; just a few publications exist on the subject; therefore, this paper represents an excellent opportunity to contribute to the evidence base in this field. Reid et al.^14^ in the preventive literature examine automated ways for detecting general retail crime using a set of fifteen visual social variables extracted from video footage from the University of Central Florida’s Crimes dataset. We were unable to locate any literature on the detection of ORC.

Our research has consequences for both theory and practice. On the theory side of things, there are two major critiques in the literature that our approach addresses: a lack of publicly available real data on which to conduct experiments and a shortage of published, well-researched methods and techniques^6^. Additionally, we accept the challenge provided by previous research, which is that future work should employ text mining techniques (in a subsequent paper). To begin, we analyze a large data set from a major marketplace platform and make the results publicly available to spur future fraud detection research in the ORC area. Second, we develop a machine learning system for detecting and preventing platform ORC. In practice, we hope to reduce fraud by identifying and pulling out bad actors or fraudsters. Specifically, we automate the finding of fraud leads in order to aid fraud investigation teams in their investigative efforts. Automation improves fraud detection and investigation efficiency, resulting in decreased operational expenses.

The rest of this paper is organized as follows: “Related work” section provides an overview of relevant literature for this topic; “The proposed framework” section provides a detailed description of the proposed framework as well as the experiments conducted in the study; “Data and methods” section offers a description of our data and methods; “Results and discussions” section provides the results and discussion; and “Conclusion and future research work” section concludes the paper and highlights opportunities for future work.

## Related work

Due to their adaptability and profitability, e-commerce platforms such as Yahoo and eBay have been increasing at a rapid pace^15^. Online fraud on these sites has increased in lockstep with this growth. The Internet Fraud Complaint Center (IFCC) has categorized online fraud into six categories: (1) non-delivery of goods; (2) product misrepresentation; (3) triangulation; (4) fee staking; (5) black-market goods sales; and (6) multiple bidding and shill bidding. Other academics have proposed various classification schemes for online fraud. For instance^16^, divide it into three time periods: pre-auction, during-auction, and post-auction, while^17^ divide it into four sorts of fraudster attitudes: aggressive, classic, luxury, and low-profile. While some research indicates that bid shielding is the most common type of fraud among these categories^7^, it is likely that different categories affect different types of online market platforms disproportionately. In our situation, we organize our materials and procedures in order to detect a certain sort of online fraud classified as (v).

In response to the growing prevalence of online fraud, researchers have developed a variety of fraud detection schemes^7^. Aleem and Antwi-Boasiako^18^ classify them into three categories: feedback anomaly detection methods, data mining schemes, and trust management schemes based on agents. Feedback anomaly detection methods employ a reputation system for the seller based on customer feedback to calculate fraud scores, with negative feedback increasing the fraud score by one and positive feedback decreasing it by one^17^. According to several researchers^18^^,^^19^ this strategy is frequently useless since it can be exploited to produce fabricated and inflated reputations. Data mining schemes are widely used today and consist of two basic steps: (1) developing features that extract user profiles and transaction histories from expertly labeled data or suspended accounts in order to discriminate between a legitimate trader and a fraudster, and (2) developing a fraud detection model based on the developed features^19,20^. Researchers frequently use a classification algorithm as the detection model. In the literature, it has been demonstrated that tree-based classification algorithms perform well^6^. Abdallah et al.^7^ summarize the most frequently used data mining techniques in the literature as follows (Table 1):

**Table 1:** Common data mining techniques in literature.

Techniques	References
Supervised learning	
Logistic regression	^21,22^
Decision trees	^19,23–28^
Artificial neural networks	^26,29^
K-nearest neighbor classifier	^20^
Bayesian classification	^30^
Support vectors machine	^28,31^
Unsupervised learning	
Association rule analysis	^32^
Clustering graph and network data (social network analysis	^24^
k-means	^20,33^
Hierarchical agglomerative clustering	^34^

Lastly, agent-based trust management solutions address issues of trust and identification through the interaction of numerous intelligent agents^21^^,^^35^.

A skewed distribution (unbalanced class) is one of the most serious problems encountered by fraud detection systems^7^. By and large, the imbalanced class issue is one in which the sample size of fraudulent instances is significantly smaller than the sample size of normal instances^36^. Working with skewed data is referred to as “imbalanced learning” Chawla et al.^37^and data in these circumstances exhibit a skewed distribution of classes in both binary and multi-class scenarios. When training traditional machine learning algorithms on imbalanced data, the minority class contributes less to the objective function minimization^38^, resulting in the model’s low performance in predicting minority class instances. In the majority of actual applications, correctly identifying minority instances is more critical^39^. Dealing effectively with this problem is essential to guaranteeing a good and robust generalization of machine learning algorithms.

Researchers have devised a variety of strategies for resolving class asymmetries, which can be classified into two broad categories: data level and algorithmic methods^7^. In the first method, balancing techniques are used to rebalance the data prior to training classification algorithms. The majority of research on fraud detection systems employs data level rebalancing strategies, which typically entail undersampling the majority class, oversampling the minority class, or a combination of the two to achieve a 1:1 ratio between the classes. Numerous proposed fraud detection systems have undersampled rather than oversampled. As the simplest form of oversampling, random oversampling does not provide additional information to the data and frequently results in model overfitting^40^. A superior alternative for oversampling is the Synthetic Minority Oversampling Technique (SMOTE)^41^. SMOTE oversamples the minority class by generating synthetic minority cases in the vicinity of the observed one. Dal Pazzolo et al.^42^ examines the SMOTE and EasyEnsemble rebalancing approaches for identifying credit card fraud and discovers that both procedures contribute to the improvement of their model outputs. As the name implies, algorithmic level methods address minority (fraudulent) classes at the algorithmic level. They include cost-sensitive learning, which assigns a cost to misclassification of the various classes based on the assumption that a cost matrix exists for the various sorts of errors^43^. Two approaches to cost-sensitive learning have been proposed in fraud detection systems: (1) metacost-thresholds, or the employment of learners who are not sensitive to class imbalance^40^, and (2) employing the learner to cope with class skewness. The learners are either intrinsically resistant to the class imbalance problem, as is the case with the Repeated Incremental Pruning to Produce Error Reduction (RIPPER) algorithm^44^ or are modified internally to be resistant to the issue, as is the case with K-nearest neighbor and support vector machine learners^7^.

In general, data-level methods outperform algorithm-level methods^7^. They are also simple to implement and have no effect on compute overhead.

## The proposed framework

**Figure 1:**
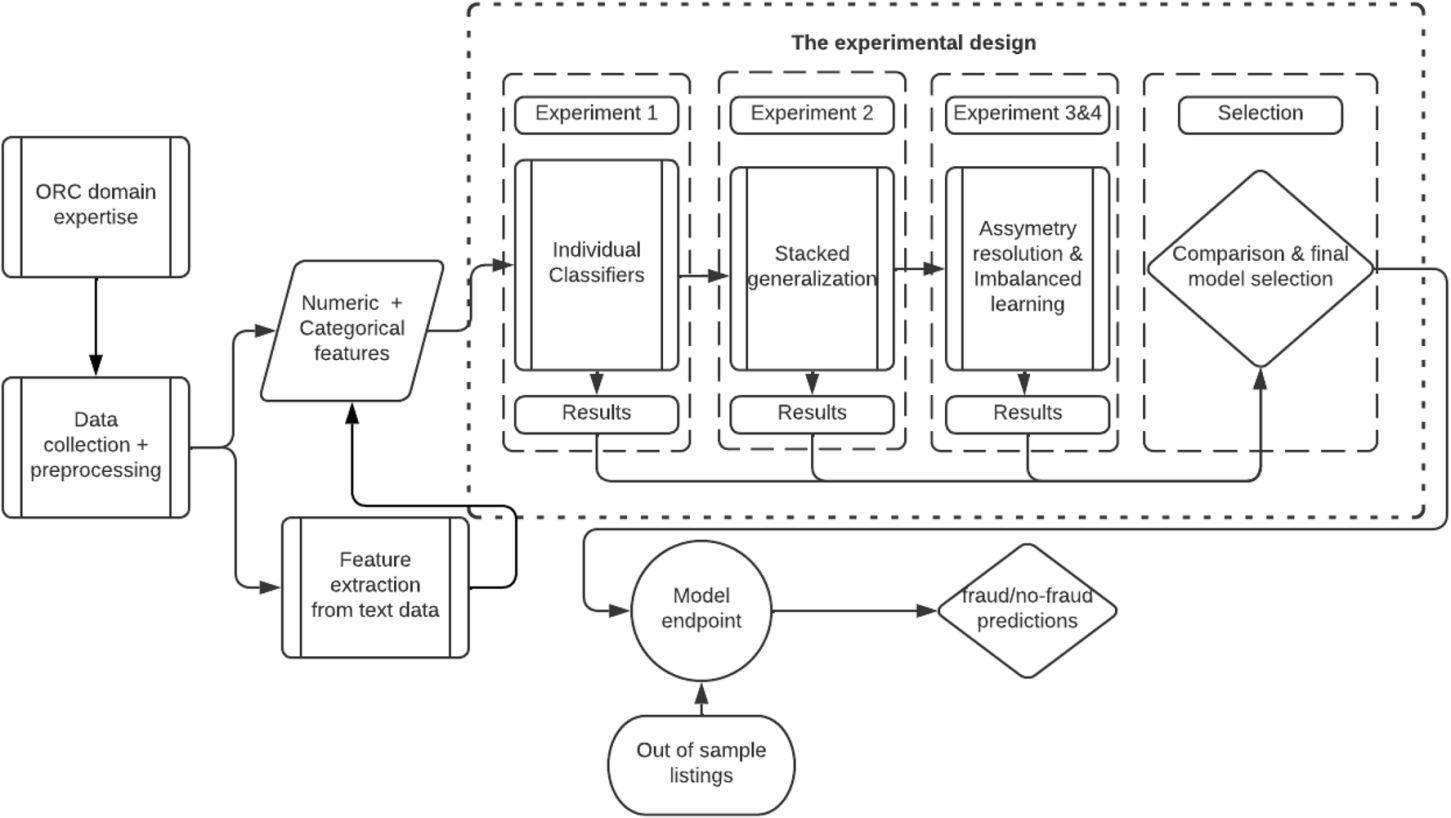
Data and information for the marketplace organized retail fraud detection system.

The proposed framework comprises four distinct experiments. When executed, the experiments lead to the identification of the best detection model for organized retail fraud instances. The dataflow diagram shown in Fig. 1 illustrates the key steps of the proposed framework.

### Experiment 1: individual classifiers

In this design, we extract numeric features and preprocess the data. Without applying any asymmetry resolution techniques, we train seven classifiers selected based on a literature review (see Table 4 for details). We use a grid search approach with repeated stratified k-fold cross validation to obtain the optimal hyperparameter configuration for each classifier. Stratification ensures that each fold of the dataset has the same proportion of observations with a given label.

### Experiment 2: stacked generalization

**Figure 2:**
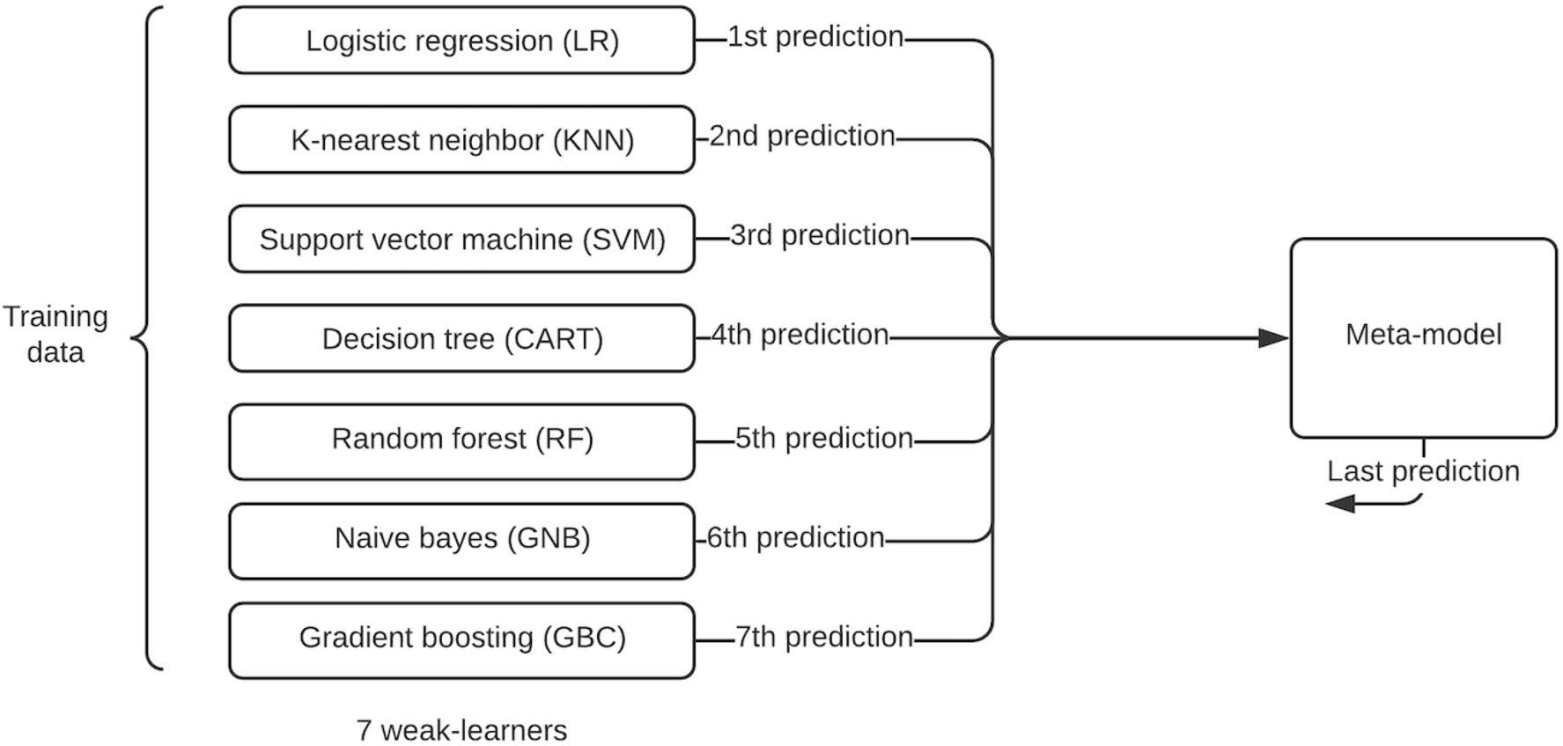
Stacked generalization approach using all the seven classifiers as weak learners.

We use the same data used in experiment 1 in this design to create an ensemble, stacking across the seven classifiers (see Fig. 2 for this architecture). This approach involves combining predictions from all the classifiers on the same data set and includes bagging and boosting. We do this to address the question of how, given multiple machine learning models that are skilled at a problem but in different ways, we can leverage the best aspects of the individual models. Generally, the architecture of a stacking model involves two or more base models, often referred to as level-0 models, and a meta-model that combines the predictions of the base models, referred to as a level-1 model. In our context, we train the meta-model on predictions made by the base models on the holdout data set. The predictions, along with the expected outputs, provide the input and output pairs of the training data set used to fit the meta-model. We follow an approach that uses k-fold cross-validation of the base models, where the out-of -fold predictions are used as the basis for the training data set. Below is a diagram to illustrate the architecture we follow:

### Experiments 3 and 4: imbalanced learning

Class asymmetry resolution is at the heart of our framework because fraud data often exhibit asymmetry in classes between fraudulent and non-fraudulent cases. As such, we search for appropriate class rebalancing techniques for our data set before repeating the steps in experiments 1 and 2 (refer to Fig. 1). Essentially, the output of this part is the best class rebalancing technique – classifier combination for our context. We describe our class resolution approach in more detail in “Data and methods” section.

## Data and methods

In this section, we present the data and the methods used in our experiments. A brief description of the classifiers is given, as are the experimental settings.

### Marketplace data

To detect the presence of ORC, we use historical data on activity and transactions from a popular worldwide online marketplace platform. We work with a sample of 3606 US-based sellers due to data labeling limits, and the primary data fields include product listing information and seller attributes. To ensure a consistent collection of listings and sellers, we restrict our research and modeling efforts to high-volume merchants (top sellers by listings within the last ninety days). The sample composition is summarized in Table 2.

**Table 2:** Marketplace sample summary.

Period	Number of listings	Number of sellers	Percentage of suspicious sellers	Variable types
November 2021 to February 2022 (90 days)	50,000	1826	7.9%	Categorical and continuous

The final data collection has a mixture of numeric, category, and text data types, with the text characteristics consisting primarily of the item’s title and description. In this paper, we rely more on the numeric and categorical features than the text features. From our data exploration, we do not find the text data to significantly improve the models’ performance. We summarize the final feature set in Table 3.

**Table 3:** Feature definitions and description.

Feature category	Feature name
Product -related	Length of the product title
	Length of the product description
	Current rice of the product
	Initial price of the product
	Product cross-posted from other groups
	Product category, for example, electronics
User-related	Age of the merchant/seller
	Number of friends of the merchant/seller
	Is the merchant/seller employee of the digital marketplace?
	Size of the largest-buyer-merchant/seller-group that the user is associated with
	Age of the account
	Median size of the buyer-merchant/seller associated with the user
Interaction-related	Lifetime number of meaningful interactions (buyer/seller interactions)
	Number of buyer reports against the merchant/seller in a day
	Number of initial messages sent in the last month
	Number of daily followers on the merchant/seller
	Number of good merchant/seller ratings received
	Number of bad merchant/seller ratings received
Product delivery-related	Promotion eligibility of the product listing
	Delivery types available for the product
	Number open customer-customer orders on the product
	Date of the latest shipped order on this product
	Date of the latest cancelled order on the product
	Product condition (new or used) as listed by merchant/seller
	Whether the product has a delivery type of shipping onsite
	Product inventory count

### Data preprocessing and feature engineering

**Figure 3:**
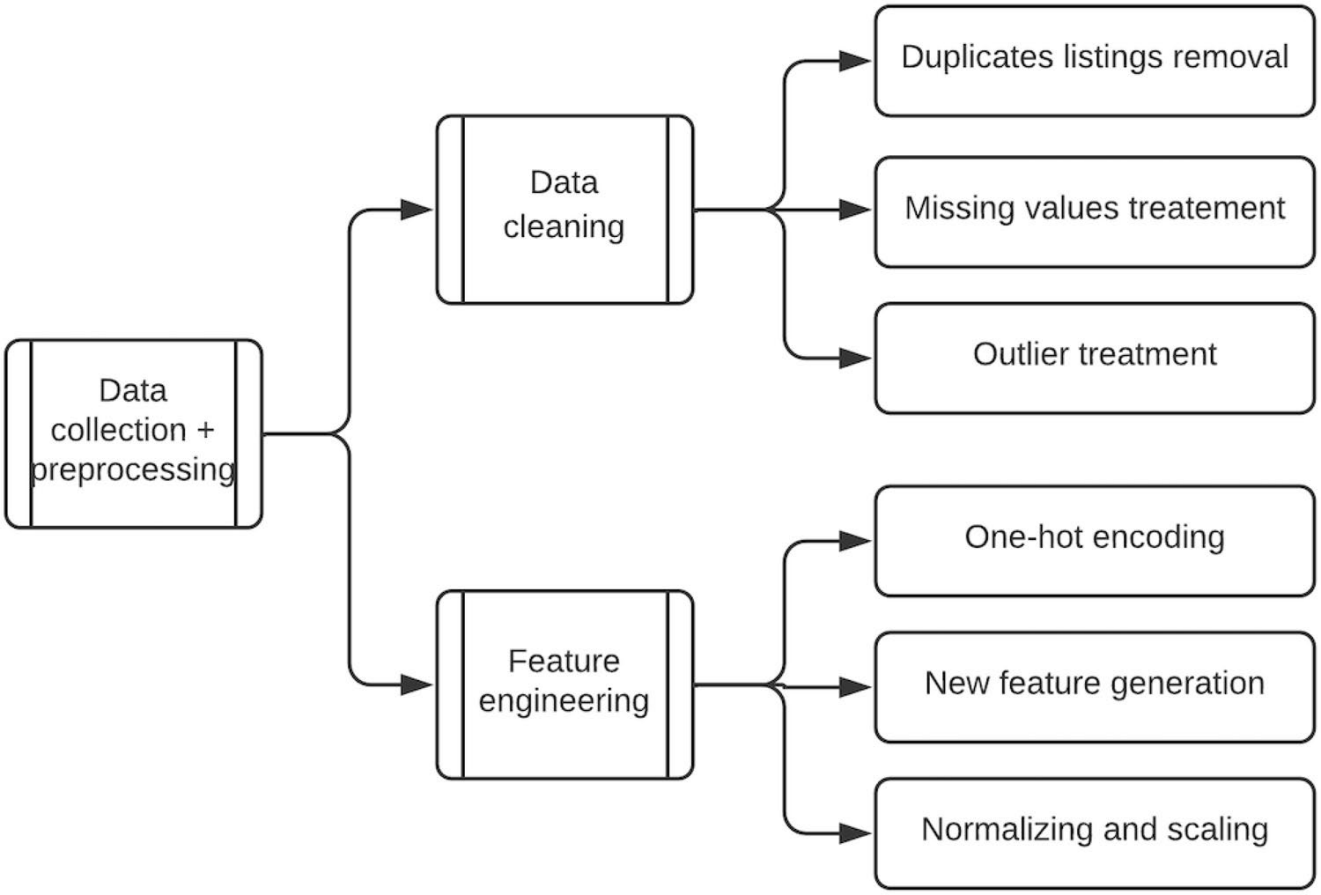
Data preprocessing steps.

As illustrated in Fig. 3 above, we undertake a number of data preprocessing operations on the dataset. They entail resolving issues such as duplicate listings, missing data, and outliers. The duplicate removal step is critical because listings can be reposted on the Marketplace; therefore, we drop duplicate listings based on the seller ID, listing title, description, and price. Missing values are handled by deleting rows or columns. If the fraction of missing data in a column is less than 20%, the concerned rows are dropped; otherwise, the entire column is dropped. We have no reason to believe that this approach diminishes the dataset’s value. We discard values that are more than three standard deviations from the mean in columns such as “product price,” where the likelihood of outlier effects is significant.

Additionally, we use feature engineering to create new predictive features from existing ones.

Our feature engineering processes include one-hot encoding categorical variables, generating dummy columns for shipping type, and generating new features based on title and product description characteristics such as the number of words, the percentage of capitalized words, and the percentage of punctuation. The final data preprocessing step entails scaling the final feature set to ensure that all features are comparable in size. In this instance, we use standard scaling. Table 3 shows a list of these features and their descriptions.

The initial selection of these features is informed by discussions with ORC experts who have extensive experience identifying and mitigating organized retail fraud cases.

### Classifiers

In the literature on fraud detection, classification techniques are frequently used to develop the detection model^6^. Classification is a supervised learning technique aimed at obtaining a discriminating function that categorizes samples^45^. Table 1 covers the most frequently used classifiers identified in the literature. We adapt these classifiers to our context as a first step in our search for the best-performing model. Additionally, we introduce new learners to improve on these baselines. We specifically add a balanced random forest classifier and a stacked ensemble of all the classifiers in our experiment. The balanced random forest classifier is designed to cope with the issue of imbalanced classes that exists in our data set. Below, we present brief descriptions of each classifier used:

#### Logistic regression

Logistic regression is similar to linear regression on classification tasks. It finds the values for coefficients $\beta_{{1,{ }}} \beta_{2} , \ldots .,\beta_{n}$ that weigh each feature $X_{1} ,{ }X_{2} ,{ } \ldots ,{ }X_{n}$ appropriately. It performs it predictions by transforming the output through a logistic function^46^. Thus, the probability of a listing being considered ORC fraud (class1) versus legitimate (class 0) can be given by:

$$P\left( {class = 1} \right) = \frac{1}{{1 + e^{ - g\left( x \right)} }}$$

where

$$g\left( x \right) = \beta_{0} + \beta_{1} X_{{1{ }}} + { }\beta_{2} X_{2} + \ldots + \beta_{n} X_{n}$$

The weights are estimated from the input data using the maximum likelihood method. If $P\left( {class = 1} \right) > 0.5$, then the listing is fraudulent, and if $P\left( {class = 1} \right) < 0.5$, the listing is legitimate.

#### K-nearest neighbor

The k-nearest neighbor algorithm assumes that similar data points are close by in n-dimensional spaces. Similarity between the data points is often measured by the distance between the points (usually the Euclidean distance or the Mahalanobis distance)^47^. The class of a new data point is predicted by a validation of the local posterior probability of each class existing by the average class membership over its k-nearest neighbors. High cardinality data sets could pose challenges for this algorithm due to it being based on the distance between data points and its dimensions^45^.

#### Support vector machine

Support-vector machines (SVMs) are supervised learning models with algorithms that analyze data for classification or regression analysis^48^. The objective of the algorithm is to find a hyperplane in an n-dimensional space that distinctly classifies the data points. The choice is based on the hyperplane that has the most significant margin, which is the hyperplane that presents the maximum distance between data points in a binary class setup. The points closest to the hyperplane are termed “support vectors” because they influence the position and orientation of the hyperplane. The number of features also influences the dimension of the hyperplane^46^.

#### Naïve bayes

This classifier makes the naive assumption that all features in the input data are independent of each other while applying Bayes’ theorem, which describes the probability of an event, based on prior knowledge of conditions that might be related to the event. More specifically, it assumes all features independently contribute to the probability of the given class, which is often a strong assumption and unrealistic in practical settings. The algorithm assumes that the off-diagonal values of the covariance matrix are zero (independent). Then the joint distribution is the product of individual univariate densities (assuming that they are Gaussian in nature)^49^.

#### Decision tree

The decision tree algorithm is a supervised learning technique that can be used to solve both classification and regression problems. It uses tree representation to solve the problem, in which each leaf node corresponds to a class label and attributes are represented on the internal node of the tree. The branch or sub-tree represents a decision rule, and the topmost node is called a decision or a root node. CART is the most commonly used type of decision tree in which classification trees are applied to a target categorical variable and the tree is used to identify the class of the target variable. Regression trees, on the other hand, are applied to a continuous target variable, and the terminal nodes of the tree contain the predicted output variable values^50^.

#### Random forest

Random forest is one of the ensemble algorithms based on boot-strap aggregation (bagging technique). Ensemble is a machine learning technique that combines several base learning algorithms in order to produce a better predictive performance model, while bagging is a technique that uses the bootstrap algorithm to obtain a random sample from a given dataset with replacement and trains the base learners and aggregates their outputs to provide a lower variance model. It creates a set of decision trees on random samples of the training data and utilizes a voting mechanism based on the predictions of each individual tree to generate a final model. During training, it selects suboptimal splits for trees by randomness of the selected subset of the training set. As a result, different models are created, and their results are combined through the voting mechanism^51^

#### Gradient boosting

Gradient boosting^52^ builds an additive model in a forward stage-wise approach. A special algorithm, two-stage logistic likelihood, is used to solve a binary classification problem:

$$L\left( {y,F} \right) = \log \left( {1 + {\text{e}}^{ - 2yF} } \right)$$

$$F\left( x \right) = \frac{1}{2}log\left( {\frac{P(y = 1|x)}{{P(y = - 1|x)}}} \right)$$

Gradient boosting of regression trees allows for the greedy optimization of arbitrary differential loss functions. At each fitting iteration, the solution (least square) tree is the one that minimizes the residuals, also known as the negative gradient of the binomial or multinomial deviance loss function. The gradient boosting method has two major parameters, the number of estimators and the learning rate. The former represents the number of boosting stages, where a large number often results in better performance, while the latter refers to a constant that controls the contribution of each tree to the model. There is often a trade-off between the learning rate and the number of estimators (n-estimators), making these two most important parameters for the algorithm.

#### Stacked generalization

Stacked generalization is an approach to minimizing the generalization error rate of one or more generalizers. With a given learning set, stacked generalization deduces the biases of the generalizers from the following steps: creating a partition of the learning set, training on one part of the partition, and then observing behavior on the other part. For a stacked model with multiple generalizers, it provides a more sophisticated strategy than the cross-validation winner-takes-all strategy for combining the individual generalizers^53^.

### Data augmentation

Our data reveal an “unbalanced data problem”, which is a term that refers to an asymmetric distribution of data across classes^38^. The majority of machine learning algorithms do not perform well on unbalanced data, as the minority cases contribute less to the objective function minimization. To address the class imbalance issue, we adapt SMOTE^37^ and its variants to our environment. It is a technique for oversampling the minority class that involves manufacturing “synthetic” examples rather than oversampling with replacement. The synthetic examples are constructed using Euclidean distances between nearest neighbors, and the process involves: (1) calculating the distance between the feature vector and its nearest neighbors; (2) multiplying this difference by a random number between 0 and 1 and adding it to the feature vector. Mathematically:

$$x_{gen} = x + \alpha \left( {x^{\prime } - x} \right)$$

The data is then balanced by continuously inserting synthetic points between minority samples and neighboring data points. This strategy effectively causes the minority class’s choice region to become more general^41^. Because SMOTE in its original form is more appropriate for numeric data, we use its variation, SMOTENC, which can deal with categorical variables, in our data. The categories of newly generated examples are determined in this variation technique by selecting the most frequent category among the nearest neighbors present throughout the generation. A completely balanced dataset generated solely by SMOTENC may not be optimal, particularly for strongly skewed class distributions with extremely sparse minority class samples, which introduces a class mixture problem. Additionally, it is necessary to clean up the noisy instances generated by interpolating between marginal outliers and inliers. To address the aforementioned difficulties, we merged SMOTENC with two under-sampling techniques: Tomek’s links (TomekLinks) and edited nearest neighbors (ENN) to improve its effectiveness in dealing with class distributions that are out of balance. A more sophisticated strategy incorporates majority under-sampling into a classifier, resulting in an ensemble model. For example, random under-sampling was integrated with boosting and bagging and applied to both classes in a tree-based method called Balanced Random Forest^54^, which provides a balanced bootstrap sample to each tree of the forest.

### The experimental setting

To conduct the fast-computing experiment, we randomly select 50 thousand rows by stratified sampling from the Marketplace listing data to ensure an unbiased representation of all subgroups. Since our experiments focus on building a fraud detection model constructed from numeric and categorical features, our first step involves developing a pipeline of these features from the listings data and matching them with marketplace account owners’ demographic, behavioral data, and transaction histories. For experiments 1 and 2, this step is followed by another pipeline that cleans the data by handling duplicates, missing values, and outliers, encodes categorical variables, and scales continuous features. In experiments 3 and 4 we add another pipeline that executes class asymmetry resolution by applying oversampling and/or undersampling techniques to create a balance between the ratios of the minority and majority classes. The final pipeline executes training, hyperparameter optimization, and evaluation of the classifiers. Tables 4 and 5 below show the hyperparameters used for tuning each classifier and the evaluation metrics applied to evaluate the performance of each classifier, respectively.

**Table 4:** The hyperparameter grid used to tuning classifiers to achieve better performance.

Classifier	Hyperparameters	Values
Logistic regression (LR)	Max_iter	500
	Classifier penalty	[none, l1, l2, ‘elastic net’]
	Classifier c	[100, 10, 1.0, 0.1, 0.01]
	Classifier solver	[‘liblinear’, ‘newton_cg’, ‘libfgs’]
k-nearest neighbor (KNN)	Number of neighbors	[1, 21]
	Metric	[‘euclidean’, ‘manhattan’, ‘minkowski’]
	Weights	[‘uniform’, ‘distance’]
Support vector machines (SVM)	Kernels	[‘linear’, ‘poly’, ‘rbf’, ‘sigmoid’]
	Gamma	
	[0.05, 0.1, 0.5, 0.7, 1]	
Decision trees (Cart)	n_estimators (# of trees)	[10, 100, 1000]
	Classifier c	[100, 10, 1.0, 0.1, 0.01]
Random forest (RF)	Max_features	[1 to 20]
	n_estimators	[10, 100, 1000]
Naïve bayes (GNB)	cv	[n_splits = 5]
Gradient boosting (GBC)	n_estimators	[1, 2, 4, 8, 16, 32, 64, 100, 200, 300, 500,1000,10000]
	Max_depth	[1, 40]
	Learning_rate	[1, 0.5, 0.25, 0.1, 0.05, 0.01]

**Table 5:** Key evaluation metrics.

Metric	Formula	Description
Accuracy (acc)	$acc=\frac{tp+tn}{tp+tn+fp+fn}$	The ratio of correct predictions by all predictions made
Precision (p)	$p=\frac{tp}{tp+fp}$	The ratio of correct positive predictions by all positively predicted classes
Recall (r)	$r=\frac{tp}{tp+fn}$	The ratio of correct positive predictions by all true positive classes
F1-score (f1)	$f1=2*\frac{p*r}{p+r}$	The harmonic mean between precision and recall

#### Hyperparameter tuning

Table 4 below shows the list of classifiers we use in our experiments and the respective hyperparameters we use to optimize their performance.

For each of the seven classifiers, the data are split into k-groups, (k = 5) in our case, where the choice of the value of k is informed by the literature review.

For each training iteration, k-1 groups of the data are used for training, while the remainder is used for validation. The groups are made, preserving the composition of the classes for our binary problem setting and each classifier is trained k times.

**Figure 4:**
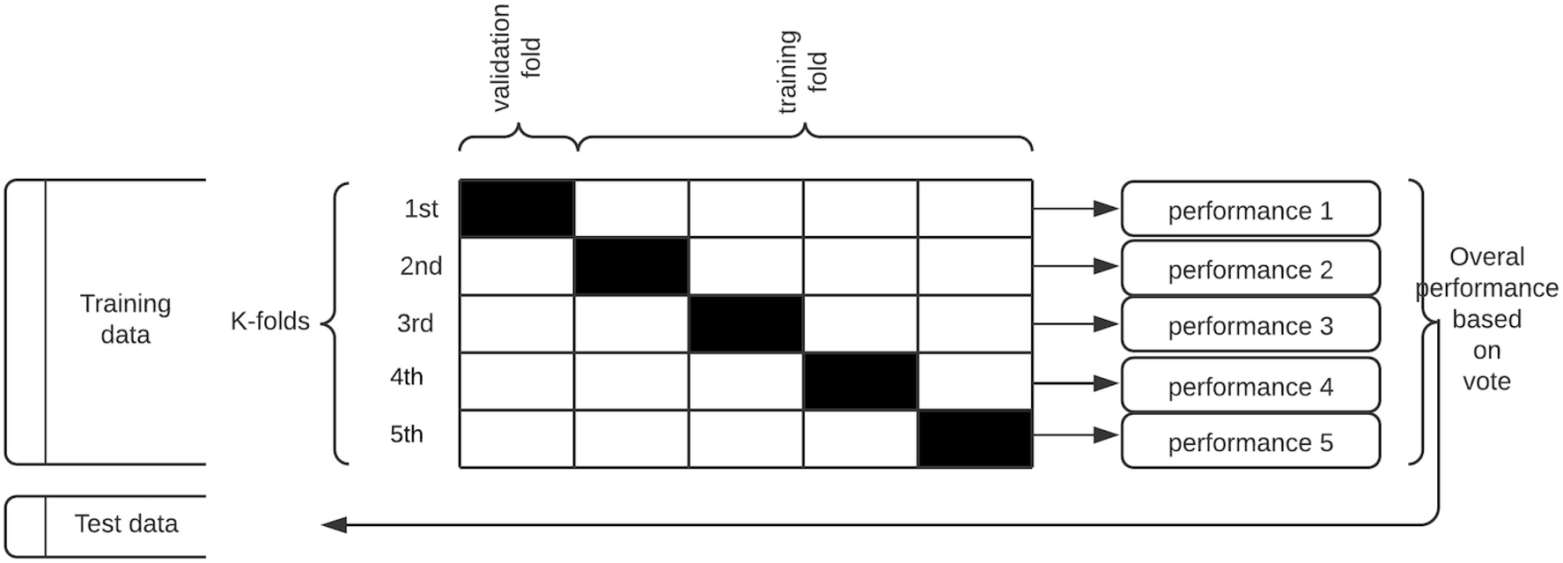
Repeated stratified k-fold cross validation procedure applied to each classification algorithm.

With k = 5, we have a fivefold cross-validation. The data are divided into 5 sets (see Fig. 4 below): set 1, set 2, set 3, set 4, and set 5. The algorithm is trained five times. In the first iteration, sets 1 through 4 are used as the training set, while set 5 is used as the validation set. In the second iteration, sets 1, 2, 3, and 5 are used as the training set and set 4 is used as the test set. This process is repeated until all the sets have been used for training and testing. The data are shuffled randomly before every split to minimize sample selection errors. The skill of each algorithm is summarized by a voting mechanism across all iterations as measured by their respective validation scores on the validation set.

The holdout set is then used to test the performance of the trained classifier in a way that mimics the production environment, as illustrated in Fig. 4 below:

Finally, we use the evaluation metrics described in below to evaluate the performance across the classifiers.

#### Evaluation metrics

Literature in this area^45^ suggests the use of the evaluation metrics listed in Table 5 below, but we pay more attention to recall, which optimizes catching bad actors and minimizes false negatives (falsely predicting suspicious listings as not suspicious). The denotations tp, tn, fp, and fn used in the formulae column below carry their regular meaning in the classification context.

Additionally, we plot ROC-AUC curves as another measure of performance. This is important because some measures, such as accuracy, are unreliable in the case of imbalanced data sets.

### Software implementation

We implement the experimental procedure based on the Python programming language using Scikit–Learn in combination with other common Python libraries such as NumPy, Pandas, Matplotlib, Seaborn and SciPy. For data acquisition and retrieval, we use structured query language (SQL) to query Hive tables where the data was initially stored.

## Results and discussions

This section summarizes and discusses the important findings from our experiments. The results are based on an 80:20 split of the data used to train and validate the classifiers. Additionally, we evaluate the classifiers’ performance using a new set of data that the classifiers have never seen before (out-of-sample test set), simulating production reality. The remainder of this part presents and discusses main results to elicit key insights that can practical application of this framework in real-world problems.

### Working with imbalanced data

A repeated stratified k-cross validation approach is used to evaluate the performance of each classifier for the unbalanced data set. Based on our evaluation metrics, we observe that although the Gaussian Naive Bayes model has the highest recall (0.954) of all models tested, including the stacked generalization model, it underperforms at predicting true positive instances and has the lowest accuracy (0.40). GNB assumes that all features are independent of one another, but given the nature of our data, this assumption may not hold true, and therefore the low results for some metrics may be explained by the violation of this critical assumption. Overall, tree-based classification models outperform others in this context, and the random forest classification model achieves the highest F1 score of all standalone models (mean value of 0.920 before hyperparameter tuning), which climbs to 0.946 after hyperparameter tuning. The RF model’s performance is consistent with the literature^7^. While these results look very promising based on in-sample validation, the true test of any classifier is best done with an out-of-sample data set. Therefore, to simulate predicting instances in the production environment, we absorb a new sample of data (never seen in training) from the marketplace platform and make predictions on it. We present the results for predictions on this out-of-sample data in Table 6 below. Our findings indicate that all classifiers experience performance degradation, albeit to varied degrees, most notably in terms of precision, recall, and F1 values. According to the literature review, we expect this kind of degradation in performance to happen because of how frequently the fraud environment changes. Fraudsters evolve their behavior to evade being caught, and therefore the fraud detection system loses its power to detect fraudulent cases over time. This finding asserts that the detection model requires regular retraining in order to detect emerging cases of fraud. Consistent with the results from the in-sample evaluation, we observe that tree-based algorithms outperform the rest.

**Table 6:** Classification results based on imbalanced data and out-of-sample performance evaluation.

Algorithm	Accuracy	Precision	Recall	F1 score	ROC-AUC
LR	0.946	0.256	0.134	0.176	0.561
KNN	0.962	0.326	0.287	0.305	0.635
CART	0.974	0.535	0.739	0.620	0.860
RF	**0.977**	**0.631**	**0.522**	**0.571**	**0.757**
SVC	0.968	0.340	0.108	0.164	0.551
GNB	0.500	0.052	0.943	0.098	0.715
GBC	0.970	0.480	0.312	0.378	0.651
SG	**0.982**	**0.665**	**0.745**	**0.703**	**0.867**

Top performing models are in bold.

### Working with balanced data

We posit that correcting for imbalanced classes in our context could help learning and ultimately the performance of our classifiers. On this premise, we proceed to apply select class rebalancing techniques based on literature and as described in “Data augmentation” section. At a high level, we test data-level and algorithmic approaches for balancing our classes. At the data-level, we test ROS, SMOTENC, SMOTENC + ENN, and SMOTENC + TomekLinks, while at the algorithmic level we test EasyEnsemble and Balanced Random Forest algorithms. Following the same evaluation approach used in the section above, we use both in and out of sample data to check the performance of each method.

**Figure 5:**
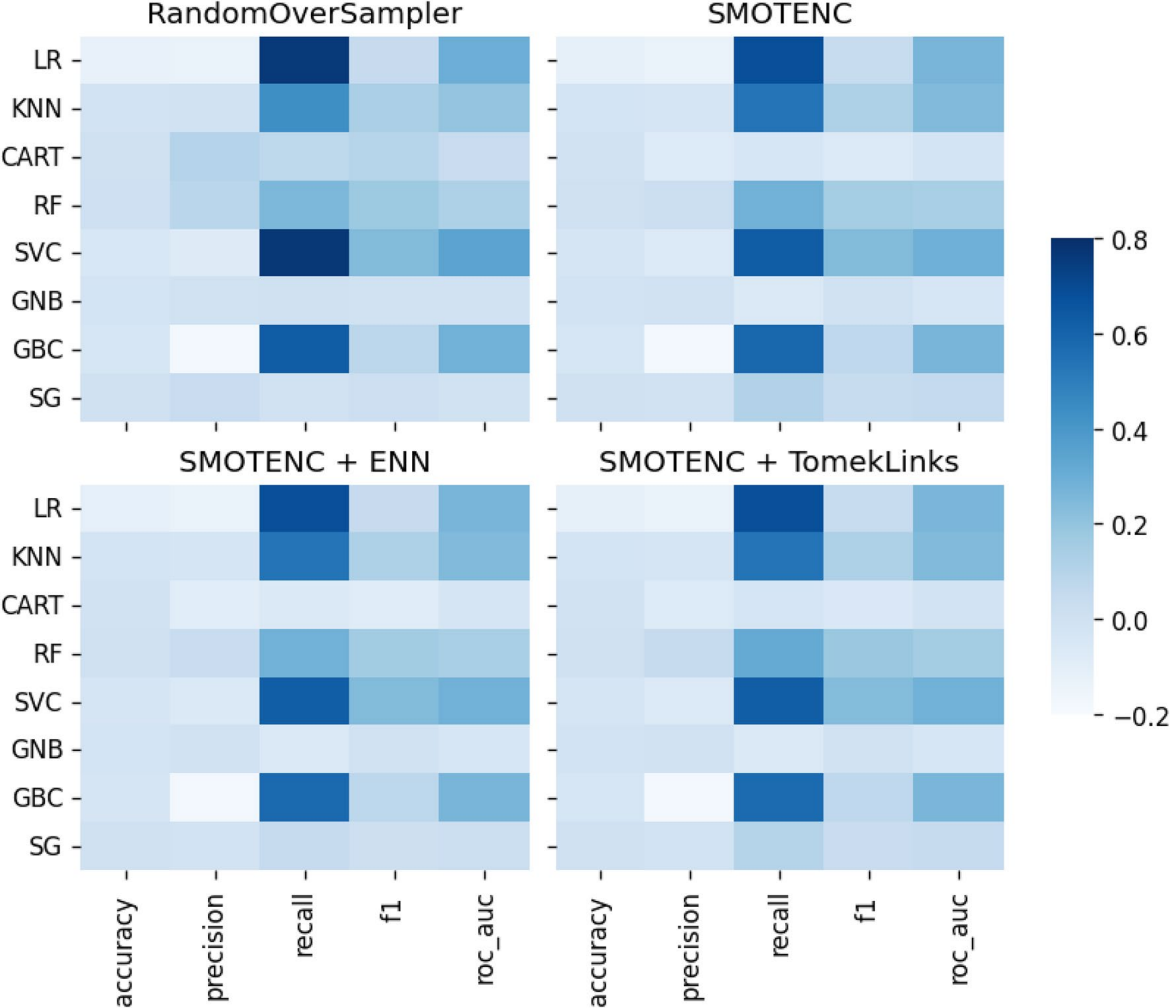
Performance improvement achieved by each classifier for a given data-level class rebalancing technique out-of-sample evaluation).

In general, our results show that the data-level approach to rebalancing classes outperforms the algorithmic approach. This finding is consistent with what we found in our literature review. Among the data-level methods, ROS outperforms all the other methods, achieving a 92.5% improvement with the in-sample set and nearly 70% with the out-of-sample set, across all the classification algorithms. SMOTENC, SMOTENC + ENN, and SMOTENC + TomekLinks achieve identical performance: 90% with in-sample data and nearly 55% with out-of-sample data. In terms of classifier–rebalancing technique combination, the Random Forest achieves the overall best performance where it registers positive improvement across all the rebalancing techniques on all performance evaluation metrics and with both in-sample and out-of-sample data. The SG classifier follows closely in overall performance. All the other classifiers register no to modest improvement across all comparison points. We show specific details of the performance in the Fig. 5 below.

As stated in our problem statement and objectives, the objective in a fraud environment is geared toward catching all the bad actors because they are the most impactful in damaging the reputation of the marketplace platform or generating losses. To that end, we err more on the side of optimizing recall values compared to the other performance metrics. With this in mind and comparing recall scores achieved through the data-level approach against the algorithmic approach, the algorithmic approach (the balanced random forest algorithm) outperforms the best data-level method–classifier combination. It achieves a top recall score of 97. 5% on in-sample data and 94.9% on out-of-sample data, against 92.8% and 81.9%, respectively. We display more details of the above discussion in Tables 7 and 8, where we show the best overall performing classifiers (RF and SG) and their various combinations with data-level class rebalancing techniques. We do this to demonstrate how they stack up against algorithmic approaches.

**Table 7:** Comparison of different class asymmetry resolution (in-sample).

Method	Modification	Sampling	Accuracy	Precision	Recall	F1 score	ROC-AUC
RandomOverSampler + RF	Data	Over-sampling	0.997 (0.001)	1.000 (0.000)	0.902 (0.023)	0.946 (0.011)	1.000 (0.000)
SMOTENC + RF	Data	Over-sampling	0.997 (0.001)	0.984 (0.010)	0.918 (0.019)	0.951 (0.012)	0.999 (0.000)
SMOTENC + ENN + RF	Data	Hybrid	0.996 (0.001)	0.985 (0.009)	0.907 (0.021)	0.944 (0.013)	0.999 (0.000)
SMOTENC + TomekLinks + RF	Data	Hybrid	0.997 (0.001)	0.985 (0.009)	0.915 (0.019)	0.949 (0.012)	0.999 (0.000)
RandomOverSampler + SG	Data	Over-sampling	0.997 (0.001)	0.996 (0.006)	0.909 (0.022)	0.951 (0.012)	0.999 (0.001)
SMOTENC + SG	Data	Over-sampling	**0.997 (0.001)**	**0.978 (0.014)**	**0.928 (0.017)**	**0.953 (0.011)**	**0.999 (0.002)**
SMOTENC + ENN + SG	Data	Hybrid	0.996 (0.001)	0.978 (0.014)	0.901 (0.021)	0.938 (0.013)	0.998 (0.001)
SMOTENC + TomekLinks + SG	Data	Hybrid	0.997 (0.001)	0.979 (0.013)	0.927 (0.018)	0.952 (0.011)	0.999 (0.001)
EasyEnsemble	Algorithm	Under-sampling	0.942 (0.004)	0.362 (0.017)	0.958 (0.015)	0.525 (0.018)	0.986 (0.005)
BalancedRandomForest	Algorithm	**Under-sampling**	**0.975 (0.003)**	**0.581 (0.027)**	**0.975 (0.012)**	**0.728 (0.022)**	**0.998 (0.001)**

Top performing models are in bold.

**Table 8:** Comparison of different class asymmetry resolution (out-of-sample set).

Method	Modification	Sampling	Accuracy	Precision	Recall	F1 score	ROC-AUC
RandomOverSampler + RF	Data	Over-sampling	0.983	0.693	0.720	0.706	0.855
SMOTENC + RF	Data	Over-sampling	**0.983**	**0.677**	**0.815**	**0.740**	**0.902**
SMOTENC + ENN + RF	Data	Hybrid	0.982	0.661	0.758	0.706	0.873
SMOTENC + TomekLinks + RF	Data	Hybrid	0.982	0.660	0.790	0.719	0.889
RandomOverSampler + SG	Data	Over-sampling	0.980	0.660	0.618	0.638	0.804
SMOTENC + SG	Data	Over-sampling	0.983	0.672	0.809	0.734	0.899
SMOTENC + ENN + SG	Data	Hybrid	0.982	0.659	0.777	0.713	0.883
SMOTENC + TomekLinks + SG	Data	Hybrid	0.982	0.648	0.796	0.714	0.892
EasyEnsemble	Algorithm	Under-sampling	0.914	0.234	0.866	0.368	0.891
BalancedRandomForest	Algorithm	Under-sampling	**0.938**	**0.312**	**0.949**	**0.469**	**0.943**

Top performing models are in bold.

Overall, we learn that in order to achieve state-of-the-art performance in this domain, important considerations have to be made during the implementation of the proposed framework. First, the choice of potential features has to be carefully made with the help of tenured domain experts. Second, machine learning algorithms consume training data in various formats, as such, appropriate preprocessing techniques have to be applied to the data before it is fed to the algorithms. The choice of the preprocessing technique depends on the input format (e.g., categorical, text, image, etc.). Feature transformation is critical in this domain. It brings efficiency to learning, the model converges faster, saving a lot of costs on compute resources. It also creates a uniform intake format and a basis for comparison across the classifiers. Third, the imbalance between classes has to be addressed. Data-level augmentation results in a more diverse set of samples and is more flexible than algorithmic-level data augmentation. Finally, organized retail fraud is a highly dynamic fraud type, therefore, once the best-performing model is selected and put into production, it should be retrained regularly to address potential drift. In “Feature importance” sectio, we provide additional details about study challenges and how we addressed them.

### Feature importance

Machine learning models can often be seen as “black box”. We take some features as input and produce some predictions as output. After training a machine learning model, we often wonder how different features affect the prediction results, what the top features are that influence the prediction results, and whether we should trust the good performance observed. Thus, model explainability plays an important role in machine learning. There are multiple techniques to explain models. In our research, we use the SHAP values approach, which is currently considered state-of-the-art machine learning model explanation technique. SHAP stands for “Shapley Additive exPlanations”. Shapley values are a commonly used approach in cooperative game theory. Essentially, they measure the contributions to the final outcome from each player separately among the coalition, while preserving the sum of contributions being equal to the final outcome. When using SHAP values in model explanation, we can measure the input features’ contribution to individual predictions. We will not cover the complex formulae used to calculate SHAP values, but more details can be found in^55^. To obtain the SHAP values of the features in our best-performing classifier, we use the SHAP Python library. Using SHAP values gives us global interpretability of our model; they not only show feature importance but also show whether the feature has a positive or negative impact on the predictions. SHAP values also provide local interpretability, giving us the opportunity to see how the features contribute to a single prediction. Other methods only show aggregated results over the whole data set.

**Figure 6:**
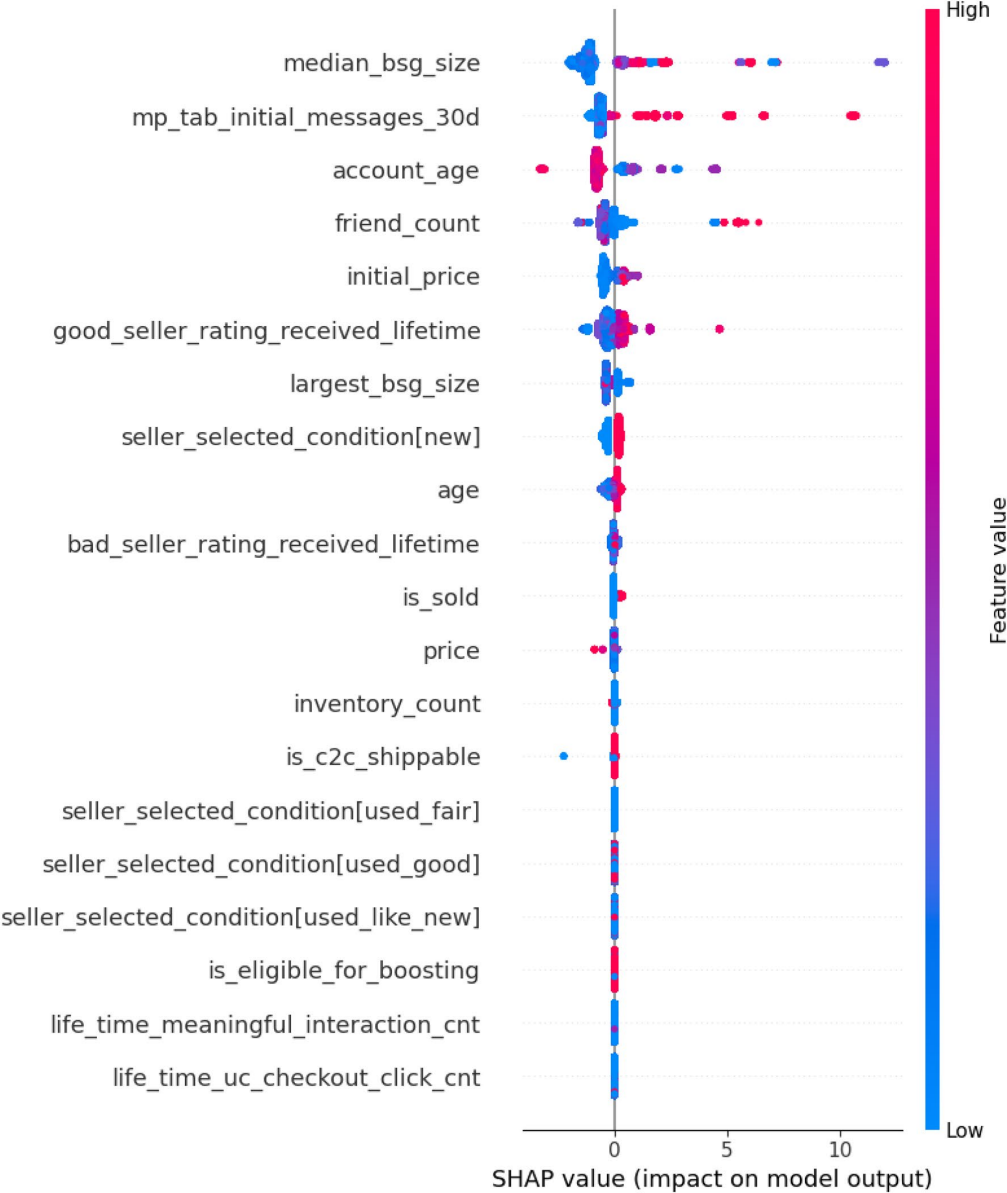
A summary of the most influential features in detect fraudulent instances.

In this research, our feature discovery efforts start with the domain experts generating the initial set of variables, potentially influential in detecting fraudulent instances. We apply these features in our experimental setting to find the best tuned classifier and class asymmetric resolution combination. Once we select the best model, we carry out an ablation analysis on it to unravel the role played by each feature. Recall, our initial features cluster around four broad groups, namely: (1) product-related, (2) user-related, (3) interaction-related, and (4) product-delivery-related features. Our analysis finds that each feature group contributes to the final list of important features. In Fig. 6, we show our features, their importance, and their range of effects over the data set.

This dot chart visualizes the directionality of the features. The x-axis shows the SHAP value (impact on model output), and the y-axis shows the names of the features. Each point on the chart is one SHAP value for a prediction and feature. Red means a higher value of a feature and blue means a lower value of a feature. For example, from the chart, we can infer that a higher value of “median_bsg_size” (Median size of the buyer-merchant/seller associated with the user) is highly associated with fraudulent prediction, and a lower value of “age” of the user is highly associated with fraudulent prediction. We can infer a general sense of the features’ directionality of impact based on the distribution of red and blue dots. Essentially, we can intuitively see how the model is using the features to make its predictions on fraudulent instances.

**Figure 7:**
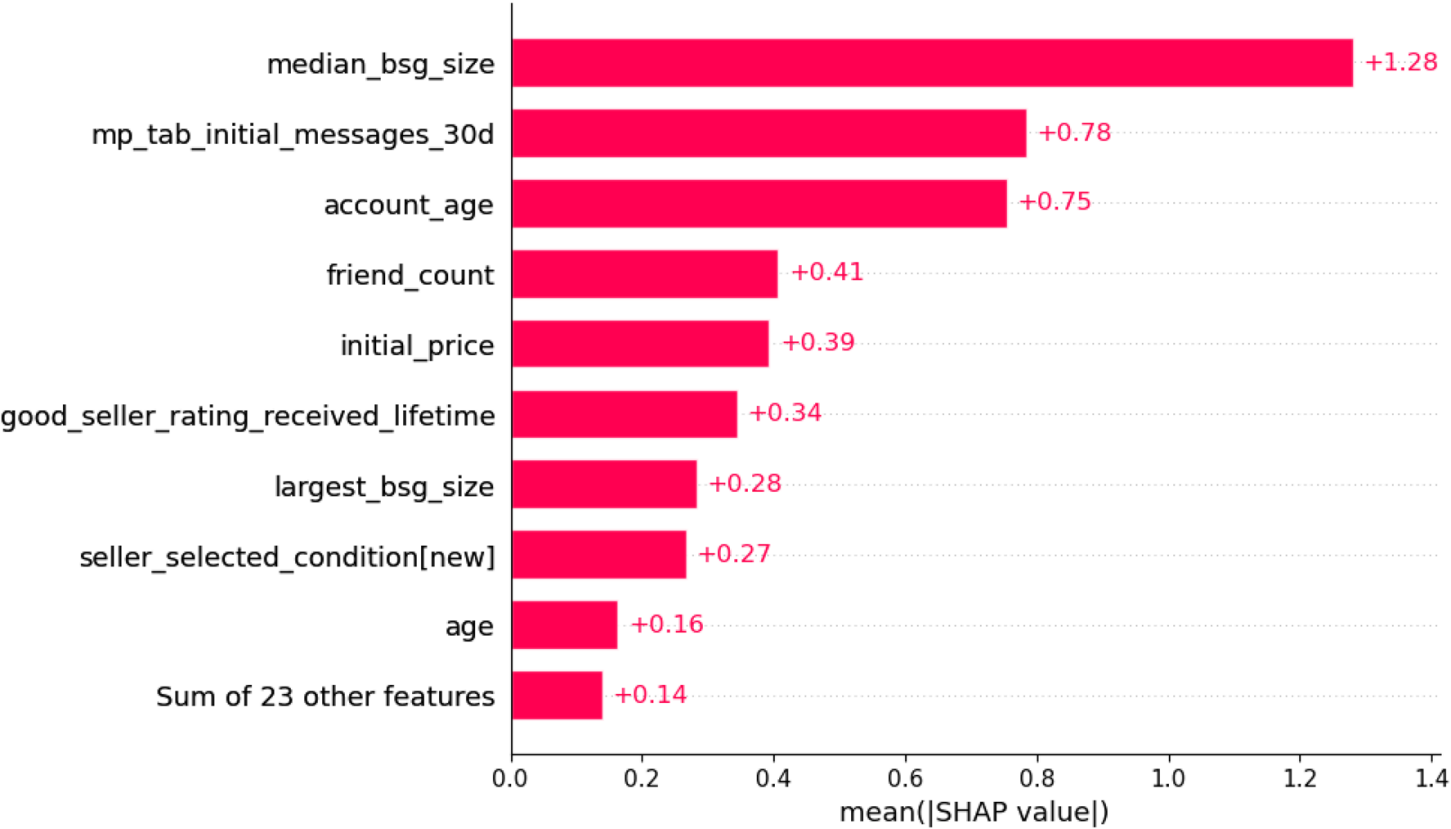
Ten most important features influencing the detection of fraudulent instances.

With the global feature importance plot in Fig. 7, we show the top ten most important features that help our model achieve state-of-the-art performance in detecting fraudulent cases. Positive SHAP value means positive impact on prediction, leading the model to predict a fraudulent instance, while negative SHAP value means negative impact, leading the model to predict a non-fraudulent case. The features are ordered by how much they influenced the model’s prediction. The x-axis shows the average of the absolute SHAP value of each feature, with higher values indicating more importance.

### Key data considerations and study limitations

Given the sequential nature of our data, we were concerned about the risk of data leakage, which occurs in machine learning when models incorporate knowledge about the data on which they were previously trained^56^. We addressed the risk of data leakage by detecting leaky features during data preprocessing using exploratory data analysis and the predictive power score matrix. We ensured temporal alignment of listings and seller features to avoid using future data in training the classifiers. Out-of-sample test data were used to evaluate the model’s performance and confirm the resolution of potential leak issues.

Limitations of our research work include known biases in the marketplace listings data, such as demographics skewed towards young individuals in the United States, and data quality issues. Omitted or improperly captured data poses challenges for data cleansing and integration. Future work could focus on investigating these problems and developing advanced data imputation methods.

Adapting filtering algorithms to evolving fraudulent activity in the e-commerce market is challenging, especially for low-volume sellers. Aggregating fraud predictions from the listing level to the seller level requires manual processes and customized rules. Continuous retraining of the automated fraud detection system is necessary to maintain performance in the face of new fraudulent behaviors^57^. Addressing data or concept drift should be considered in future implementations to tackle performance issues.

Our initial feature set is based on conversations with industry experts on organized retail fraud, primarily from the North American region. While efforts were made to mitigate regional information bias, some biases may still exist due to variations in text composition and semantics across regions. However, we mitigate this limitation by using high-level physical attributes of the title and description of the listing. Exploratory results indicate that listings with higher numbers of characters are more likely to contain organized retail fraud products, aligning with previous research findings^58^.

## Conclusion and future research work

Retail organized crime has been a persistent cybersecurity issue for e-commerce platforms such as Meta’s Marketplace and eBay, among others. With the growing amount of data available on users’ attributes and transaction histories, it’s becoming increasingly difficult to spot fraudulent actions using filtering rules and key word search and refinement. In our research, we proposed an automated fraud detection method for detecting possible frauds in the organized retail crime space using a supervised machine learning approach. We demonstrated that our system outperformed past systems based on rule-based and unsupervised learning approaches in terms of prediction accuracy and efficacy. To the best of our knowledge, this approach has not been applied in ORC settings, and where it has been applied in other contexts, majority of cases have only used single-stage trials for data processing and/or imbalance learning. In our case, we demonstrated how to optimize a fraud detection modeling system by combining expert informed feature discovery, bespoke data processing, imbalanced learning, feature, and model selection, customized hyperparameter setup, and business-oriented assessment metrics to achieve state-of-the-art performance. In this work, we mainly used numeric and categorical features. Future work can focus on using a multimodal feature set (combination of numeric, text and image data) to train the algorithms. These additional features could potentially achieve a higher or similar performance without necessarily relying heavily on ORC domain experts.

## Data Availability

The data sets generated and/or analyzed during the current study are not publicly available due to sensitive information but are available from the corresponding author on reasonable request.

## Abbreviations

ML: Machine learning
ORC: Organized retail crime
RTC: Retail theft cases
LR: Logistic regression
KNN: k-nearest neighbor
SVM: Support vector machine
CART: Classification and regression tree
RF: Random forest
GNB: Gaussian naive bayes
GB: Gradient boosting
BRF: Balanced random forest
SG: Stacked generalization
FDM: Fraud detection model
SMOTE: Synthetic minority oversampling technique
SMOTENC: Synthetic minority oversampling technique for nominal and continuous
CV: Cross validation
EDA: Exploratory data analysis
TP: True positive
TN: True negative
FP: False positive
FN: False negative
